# Upadacitinib in Patients With Difficult-to-Treat Crohn’s Disease

**DOI:** 10.1093/crocol/otae060

**Published:** 2024-10-24

**Authors:** Cristina Bezzio, Gianluca Franchellucci, Edoardo V Savarino, Mauro Mastronardi, Flavio Andrea Caprioli, Giorgia Bodini, Angela Variola, Franco Scaldaferri, Federica Furfaro, Emma Calabrese, Maria Beatrice Principi, Giuseppe Biscaglia, Manuela Marzo, Andrea Michielan, Carolina Cavalli, Annalisa Aratari, Michele Campigotto, Linda Ceccarelli, Maria Cappello, Simone Saibeni, Paola Balestrieri, Alessandra Soriano, Valentina Casini, Lorenzo Bertani, Brigida Barberio, Francesco Simone Conforti, Silvio Danese, Alessandro Armuzzi

**Affiliations:** Gastroenterology Department, IBD Unit, IRCCS Humanitas Research Hospital, Rozzano, Italy; Department of Biomedical Sciences, Humanitas University, Pieve Emanuele, Italy; Gastroenterology Department, IBD Unit, IRCCS Humanitas Research Hospital, Rozzano, Italy; Department of Biomedical Sciences, Humanitas University, Pieve Emanuele, Italy; Division of Gastroenterology, Department of Surgery, Oncology and Gastroenterology, University of Padua, Padua, Italy; Department of Gastroenterology, S. de Bellis National Institute of Gastroenterology, Castellana Grotte, Italy; Gastroenterology and Endoscopy Unit, Fondazione IRCCS Ca’ Granda Ospedale Maggiore Policlinico, Milan, Italy; Department of Pathophysiology and Transplantation, Università degli Studi di Milano, Milan, Italy; Department of Internal Medicine, IRCCS Policlinico San Martino, Università di Genova, Genoa, Italy; IBD Unit, IRCCS Ospedale Sacro Cuore Don Calabria, Negrar di Valpolicella, Italy; IBD Unit, CEMAD, Digestive Diseases Center, Medicina Interna e Gastroenterologia. Dipartimento di Scienze Mediche e Chirurgiche, Fondazione Policlinico Universitario “A. Gemelli” IRCCS, Rome, Italy; Department of Gastroenterology and Endoscopy, IRCCS San Raffaele Hospital, Vita-Salute San Raffaele University, Milan, Italy; Department of Systems Medicine, Gastroenterology Unit, University of Rome “Tor Vergata,” Rome, Italy; Department of Precision and Regenerative Medicine and Ionian Area (DiMePRe-J), Gastroenterology Unit, University of Bari “Aldo Moro,” Bari (BA), Italy; Division of Gastroenterology, Fondazione IRCCS “Casa Sollievo della Sofferenza,” San Giovanni Rotondo, Italy; Division of Gastroenterology, Veris-Delli Ponti Hospital, Scorrano, Italy; Azienda Provinciale per i Servizi Sanitari (APSS), Gastroenterology and Digestive Endoscopy Unit, Santa Chiara Hospital, Trento, Italy; Gastroenterology Unit, Santa Maria Degli Angeli Hospital, Pordenone, Italy; IBD Unit, S. Filippo Neri Hospital, Rome, Italy; Department of Medical, Surgical and Health Sciences, University of Trieste, Trieste, Italy; Department of Translational Sciences and New Technologies in Medicine and Surgery, Gastrointestinal Unit, University of Pisa, Pisa, Italy; ProMISE Department, Gastroenterology and Hepatology Section, University of Palermo, Palermo, Italy; Gastroenterology Unit, Rho Hospital, ASST Rhodense, Rho, Italy; Unit of Gastroenterology and Digestive Endoscopy, Campus Bio-Medico University, Rome, Italy; Gastroenterology Division, Internal Medicine Department, Azienda Unità Sanitaria Locale-IRCCS, Reggio Emilia, Italy; Unit of Gastroenterology and Digestive Endoscopy, ASST Bergamo Est, Bolognini Hospital, Seriate (BG), Italy; Department of General Surgery and Gastroenterology, Tuscany Northwest ASL-Pontedera Hospital, Pontedera, Italy; Division of Gastroenterology, Department of Surgery, Oncology and Gastroenterology, University of Padua, Padua, Italy; Gastroenterology and Endoscopy Unit, Fondazione IRCCS Ca’ Granda Ospedale Maggiore Policlinico, Milan, Italy; Department of Gastroenterology and Endoscopy, IRCCS San Raffaele Hospital, Vita-Salute San Raffaele University, Milan, Italy; Gastroenterology Department, IBD Unit, IRCCS Humanitas Research Hospital, Rozzano, Italy; Department of Biomedical Sciences, Humanitas University, Pieve Emanuele, Italy

**Keywords:** upadacitinib, Crohn’s disease, difficult-to-treat IBD, effectiveness, remission, response

## Introduction

Crohn’s disease (CD) is characterized by a relapsing-remitting course that causes progressive bowel damage with a risk of stricturing and penetrating complications and severely impairs patients’ quality of life.^1^ Despite an expanding array of drugs, including biologics, for CD therapy, approximately one-third of patients do not respond to the initial treatment, and half of them lose the response over time.^2^

Recently, upadacitinib, a selective Janus kinase 1 inhibitor, has been approved to treat adults with moderate-to-severe active CD. Upadacitinib showed efficacy in the management of disease activity and mucosal healing in the U-EXCEED and U-EXCEL induction trials and ENDURE maintenance study, where the coprimary endpoints (clinical remission and endoscopic response) and key secondary endpoints were successfully achieved.^3^ The clinical remission rates in these trials ranged from 38.9% to 49.5% at 12 weeks. Given its efficacy and favorable benefit-risk profile, upadacitinib offers substantial added value in the treatment of CD, in both patients with previous failure of immunosuppressants or biologics and biologic-naive patients.^4^ So far, 2 studies examined the effectiveness and safety of upadacitinib in real-world settings,^5,6^ but not in patients with difficult-to-treat CD. Therefore, this study assessed the effectiveness and safety of upadacitinib in CD patients in whom all other therapies had failed.

### Patients and Methods

This observational cohort study was conducted in tertiary centers in Italy, where upadacitinib became prescribable on a compassionate-use basis in 2023. Compassionate use was allowed for adults with CD when all other reimbursed therapies were ineffective and only with ethics committee approval. The study was approved by the ethics committee of the coordinating center, IRCCS Humanitas Research Hospital (Rozzano, Italy).

Patients with moderate-severe CD (defined as Harvey-Bradshaw index [HBI] > 8 or Simple endoscopic score for Crohn’s disease [SES-CD] > 6), who had received upadacitinib after exhausting other treatments were included. Crohn’s disease was diagnosed according to ECCO guidelines.^7^ Inclusion criteria were age ≥ 18 and a follow-up of at least 6 months. Exclusion criteria were a medical history of venous thromboembolic or acute arterial events and cardiovascular risk factors. The upadacitinib dosing schedule was 45 mg/day for 12 weeks (induction), followed by 30 mg/day for another 12 weeks (maintenance).

The following data were collected from medical charts at baseline: age, age at CD diagnosis, sex, current smoking habit, disease location, behavior and activity, previous and concomitant medications, previous CD-related abdominal surgery, and extra-intestinal manifestations. At baseline and at 12 and 24 weeks, data were also collected on: HBI,^8^ use of corticosteroids, serum C-reactive protein, fecal calprotectin, ultrasonographic features, need for surgery, and adverse events.

### Clinical Endpoints

The primary endpoint was corticosteroid-free clinical remission, defined as HBI ≤ 3, after 12 weeks of therapy. Secondary endpoints at 12 weeks were: clinical response (decrease in HBI > 3 points); biochemical remission (fecal calprotectin < 150 μg/g and C-reactive protein < 0.5 mg/dL); transmural response (reduction of bowel wall thickness by 2 mm); transmural healing (bowel wall thickness < 3 mm); deep remission (clinical remission, biochemical remission, and transmural healing); and continuation of therapy.

## Results

The study included 64 CD patients with a mean disease duration of 14.5 years (Table 1).

**Table 1.** Clinical characteristics of 64 patients with difficult-to-treat Crohn’s disease, at baseline.

Characteristic	Value
Age, years, mean (SD)	64.2 (9.8)
Age disease at diagnosis, years, mean (SD)	22.4 (9.8)
Disease duration, years, mean (SD)	14.5 (9.2)
Female, *n* (%)	25 (39.1)
Current smokers, *n* (%)	10 (15.6)
Previous abdominal surgery, *n* (%)	43 (67.2)
Extra-intestinal manifestations, *n* (%)	28 (43.8)
Disease location, *n* (%)	
Ileal	10 (15.6)
Colonic	16 (25.0)
Ileocolonic	38 (59.4)
Upper disease, *n* (%)	11 (17.2)
Perianal disease, *n* (%)	29 (45.3)
Current use of corticosteroids, *n* (%)	29 (45.3)
Behavior, *n* (%)	
Inflammatory	26 (40.6)
Stricturing	21 (32.8)
Fistulizing	17 (25.6)
Clinical disease activity, *n* (%)	
Mild (HBI^c^ 5–7)	5 (7.9)
Moderate (HBI^c^ 8–16)	46 (73.0)
Severe (HBI^c^ > 16)	12 (19.0)
Endoscopic disease activity, *n* (%)	
Mild (SES-CD^d^ < 6)	0 (0.0)
Moderate (SES-CD^d^ 6–15)	36 (56.2)
Severe (SES-CD^d^ > 15)	28 (43.8)
C-reactive protein > 0.5 mg/dL, *n* (%)	38 (59.4)^a^
Fecal calprotectin > 250 μg/g feces, *n* (%)	50 (86.2)^b^
Fecal calprotectin > 150 μg/g feces, *n* (%)	58 (100)^b^

^a^Data missing for 1 patient.

^b^Data missing for 6 patients.

^c^HBI, Harvey-Bradshaw index.

^d^SES-CD, simple endoscopic score for Crohn’s disease.

All patients had failed all approved and reimbursed medications for the treatment of Crohn’s disease in Italy (corticosteroids, thiopurines, infliximab, adalimumab, vedolizumab, and ustekinumab). Prior to receiving upadacitinib, 43 patients had undergone at least 1 surgical procedure (30 ileocecal, 8 Jejunoileal, and 5 colonic resections). A history of extra-intestinal manifestations was present in 28 patients, including 17 with peripheral arthritis, 5 with axial arthritis, 2 with pyoderma gangrenosum, 3 with erythema nodosum, and 1 with uveitis. Disease location was ileocolonic in 38 cases, concomitant upper disease was registered in 11 cases, and 29 patients had concomitant perianal disease. Clinical disease activity was present in all cases (46 moderate and 12 severe), and 29 patients (45.3%) were on corticosteroid therapy. A disease extension > 30 cm was found in 16 cases. Patients with perianal disease localization were 29; however, of whom only 10 had active perianal disease at the time of enrollment.

### Effectiveness After 12 Weeks

**Figure 1.**
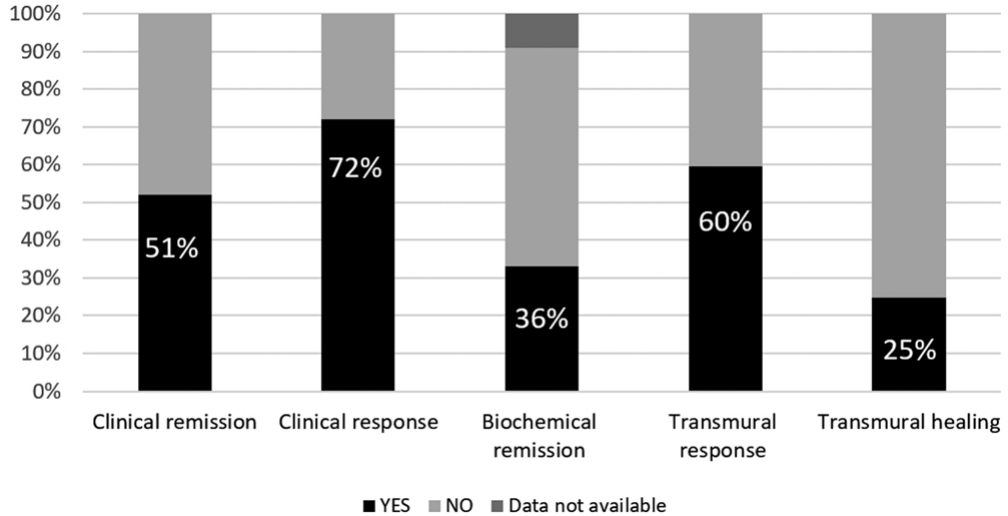
Clinical endpoints after 12 weeks of therapy with upadacitinib. The primary endpoint was clinical remission (Harvey-Bradshaw index ≤ 3).

After 12 weeks of therapy, steroid-free clinical remission was achieved in 33 patients, and a clinical response was observed in 46 patients (Figure 1). The mean HBI decreased from 11.90 (SD = 3.61) to 4.62 (SD = 4.90). Among the 58 patients with fecal calprotectin and C-reactive protein data at both baseline and 12 weeks, biochemical remission occurred in 21 cases (36.2%).

Ultrasonographic assessment was done at baseline and 12 weeks in 52 patients. At baseline, all patients had pathological thickening (>3 mm) of an intestinal segment; the most commonly involved intestinal segment was the ileum (43 patients) followed by the colon (21 patients). Transmural healing occurred in 15 patients (28.8%), and a transmural response occurred in 38 patients (73.0%). Overall, deep remission occurred in 14 (21.8%) of the 64 patients.

Therapy was discontinued before the end of the 12-week induction period in 14 patients (21.9%) because of therapy failure (9 cases) or adverse events (5 cases). During the induction period, 6 patients (9.4%) had surgery for intestinal perforation (1 case) or persistent disease activity (5 cases). After surgery, 3 patients resumed upadacitinib, while the others changed therapy.

### Effectiveness After 24 Weeks

Considering the 50 patients who maintained therapy with upadacitinib after 12 weeks, clinical remission was achieved at 24 weeks in 39 patients, and a clinical response was observed in 48 patients. Among the 60 patients with fecal calprotectin values at baseline and after 12 or 24 weeks of upadacitinib, biochemical remission occurred in 23 (38.3%). After 22 weeks, 1 patient temporarily stopped therapy due to the onset of acute arthritis, despite being in clinical remission. Of the 10 patients with active perianal disease at baseline, 7 reported a reduction in fistula drainage, and 2 reported no change at the end of follow-up period. None of the 29 patients with a history of perianal disease, reported a worsening or reactivation of perianal disease.

### Adverse Events

During the induction period, 14 adverse events (in 13 patients) were reported. Of these, 7 led to the temporary suspension or discontinuation of upadacitinib. Temporary suspension was due to *Bordetella pertussis* infection and liver abscess (one case each); when these conditions resolved, the patients resumed upadacitinib therapy. Permanent discontinuation was due to severe acute kidney injury (creatinine, 6.5 mg/dL), herpes zoster, hepatitis (AST and ALT > 500 IU/mL), bowel perforation, and severe anemia (Hb < 8.0 g/dL). The dosage of upadacitinib was never reduced. In case of a serious adverse event or an adverse event due to the drug, the therapy was discontinued. The remaining adverse events were mild. One patient had an increase in cholestasis markers (GGT and ALP > 1.5 times the upper limit of normal) and bacterial pharyngitis. Another patient had an increase in creatine kinase without signs or symptoms of rhabdomyolysis. Two cases each had folliculitis and arthralgia. During the maintenance period, 1 patient suspended therapy for acute arthritis.

## Discussion

In this study, 64 CD patients in whom all available treatments had failed received upadacitinib on a compassionate use basis. After 12 weeks of upadacitinib (45 mg/day), steroid-free clinical remission (the primary endpoint) was achieved in 33 cases (51.6%) and a clinical response was observed in 46 (71.8%). Notably, biochemical remission occurred in 21 (36.2%) of the 58 patients for whom this endpoint was determined. Transmural healing was achieved in 15 of 52 patients (28.8%), and a transmural response was observed in 38 of 52 patients (73.4%). Deep remission occurred in 14 (21.8%) of the 64 patients overall.

Recently, a consensus has determined that difficult-to-treat Inflammatory Bowel Disease (IBD) can be diagnosed after the failure of biologics and advanced small molecules with at least 2 different mechanisms of action.^9^ This condition is met in a high percentage of CD patients who have persistent disease activity despite medical and surgical treatments. This study included the most severe cases of CD, mostly patients who had already undergone surgery and were refractory to all possible biological therapies for the disease. Despite this, the rates of clinical remission and clinical response, as well as the speed of action, are comparable to those of registration trials.

There are few real-world data on the effectiveness and safety of upadacitinib in CD. A retrospective cohort study of 33 patients had a clinical response rate of 69.7% and remission rate of 27.2% at 12 weeks.^5^ A prospective study of patients with CD or ulcerative colitis observed a clinical response in 13 of 17 CD patients (76.5%) and clinical remission in 12 CD patients (70.6%) in an 8-week period.^6^

Our results are similar despite variations in upadacitinib dosage and inclusion criteria across studies (eg, our study included only patients refractory to Crohn’s disease therapies). This suggests that upadacitinib may have comparable efficacy in difficult-to-treat patients and those patients with different clinical history or clinical features.

In our study, 13 patients had a drug-related adverse event and in 7 cases, the drug was temporarily or permanently discontinued. Thus, our study demonstrates an acceptable safety profile of upadacitinib, considering the clinical characteristics of the study population (failure of all biological CD therapies, stenosing/fistulizing disease, and a high rate of abdominal surgery). In the U-EXCEED, U-EXCEL, and CELEST^10^ trials, the rates of drug discontinuation were close to 10% in both the 15 and 30 mg groups.^3^ The previously cited prospective real-world study^6^ reported a 5.7% rate of therapy discontinuation due to drug-related adverse events, considering both ulcerative colitis and CD patients, with only 1 discontinuation among CD patients. Notably, our study did not record deep vein thrombosis or cardiovascular events, although 1 case each of herpes zoster and *B. pertussis* infection occurred in patients who refused vaccination prior to upadacitinib exposure. Additionally, 1 case of perforation was noted, consistent with findings from registration trials.^3^

This study has some limitations: it was retrospective with a short, 24-week follow-up, and data for 3 secondary endpoints (biochemical remission, transmural healing, and transmural response) were missing for 4–10 patients. The data are not generalizable to all patients with Crohn’s disease but only to the subgroup of difficult-to-treat patients, who are primarily managed in tertiary referral centers for IBD care. Nonetheless, this multicenter study collected useful effectiveness and safety data on upadacitinib in patients with severe CD (high HBI at baseline and previous abdominal surgery in 67.2% of cases) who were refractory to all biological drugs when administered at the correct dosage for CD.

Overall, this study underscores the efficacy of upadacitinib in CD patients in whom multiple drugs have failed, and it suggests that upadacitinib is effective even in patients with difficult-to-treat CD.

## Data Availability

Data not publicly available. The datasets used and/or analyzed during the current study are available from the corresponding author on reasonable request.
