# Correction to: Upadacitinib in Patients With Difficult-to-Treat Crohn’s Disease

**DOI:** 10.1093/crocol/otaf022

**Published:** 2025-03-25

This is a correction to: Cristina Bezzio, Gianluca Franchellucci, Edoardo V Savarino, Mauro Mastronardi, Flavio Andrea Caprioli, Giorgia Bodini, Angela Variola, Franco Scaldaferri, Federica Furfaro, Emma Calabrese, Maria Beatrice Principi, Giuseppe Biscaglia, Manuela Marzo, Andrea Michielan, Carolina Cavalli, Annalisa Aratari, Michele Campigotto, Linda Ceccarelli, Maria Cappello, Simone Saibeni, Paola Balestrieri, Alessandra Soriano, Valentina Casini, Lorenzo Bertani, Brigida Barberio, Francesco Simone Conforti, Silvio Danese, Alessandro Armuzzi, Upadacitinib in Patients With Difficult-to-Treat Crohn’s Disease, *Crohn’s & Colitis 360*, Volume 6, Issue 4, October 2024, otae060, https://doi.org/10.1093/crocol/otae060

The following changes have been made to the originally published manuscript. The **Funding** section statement has been changed to read: “This work was partially supported by Ricerca Corrente funding from Italian Ministry of Health to IRCCS Humanitas Research Hospital.” instead of “None declared.”

